# Utilisation of a Novel Test to Measure Severity and Treatment Efficacy of Posterior Blepharitis

**DOI:** 10.1155/2015/617019

**Published:** 2015-08-12

**Authors:** Steven T. H. Yun, David M. Woo, Calum W. K. Chong, Ying Liu, Katherine E. Francis, Saumil A. Shah, Ashish Agar, Ian C. Francis

**Affiliations:** ^1^The Department of Ophthalmology, Prince of Wales Hospital, Sydney, NSW 2031, Australia; ^2^The University of New South Wales, Sydney, NSW 2052, Australia

## Abstract

*Background*. This study evaluated the effectiveness of managing posterior blepharitis (PB) using a novel Posterior Blepharitis Management Protocol (PBMP).* Design*. Prospective, consecutive case series with 100% followup to one month.* Participants*. 27 patients (54 eyes) with PB from an Ophthalmology practice in Sydney, Australia.* Methods*. Each patient's PB was assessed by grading the nature and expressibility of the central lower lid tarsal gland secretions on Compression Of The Eyelid (COTE). Patients were then instructed in detail to undertake daily PB management sessions at home using our modified PBMP.* Main Outcome Measures*. On a subjective scale, patients compared their symptoms at one month with baseline. COTE scores were reevaluated to assess the objective effectiveness of each individual's PBMP. COTE scoring was described as grades 1 (clear oil), 2 (pus, liquid), 3 (toothpaste-like secretions), and 4 (complete tarsal gland obstruction).* Results*. Patients reported a mean 77.8% ± 13.5% subjective improvement in symptoms. There was a trend towards improvement in COTE grading at one month compared with baseline: grades 1 (0 to 7.4%), 2a (22.2 to 16.6%), 2b (7.4 to 3.7%), 3 (18.5 to 27.7%), and 4 (51.8 to 44%).* Conclusions*. PBMP provided a rapid, inexpensive, simple, effective, and safe method of treating PB.

## 1. Introduction

Posterior blepharitis (PB) is a common, chronic, and potentially sight-threatening eyelid and ocular surface disease, characterised by inflammation and obstruction of the meibomian glands [1, 2]. Symptoms include ocular surface discomfort typically worse in the mornings, as well as tearing, grittiness, photophobia, and blurred vision [3]. The signs of PB include red lid margins, increased visibility of the meibomian orifices, lash loss, prominent visible tarsal glands, and changes in tarsal gland expressibility.

While PB is frequently seen, its prevalence is difficult to determine because of the lack of a standardised classification of severity [4, 5]. Moreover, it is clear that for many patients, management of PB can be prolonged, ineffective, and frustrating [6].

The last International MGD Workshop (2011) summarised various grading systems used to assess MGD, focusing on meibum expressibility and quality [7]. Hitherto, none has been adopted as a gold standard.

This study evaluated PB both subjectively and objectively. Objective assessment of PB was performed using the Compression Of The Eyelid (COTE) grading system (Table 1). A novel technique, the Posterior Blepharitis Management Protocol (PBMP), was applied for one month and its outcomes were assessed.

## 2. Design

Twenty-seven patients (54 eyes) with clinically confirmed PB from a general Ophthalmology practice in Sydney, Australia, were enrolled in this prospective, consecutive case series.

### 2.1. The COTE Test

**Figure 1:**
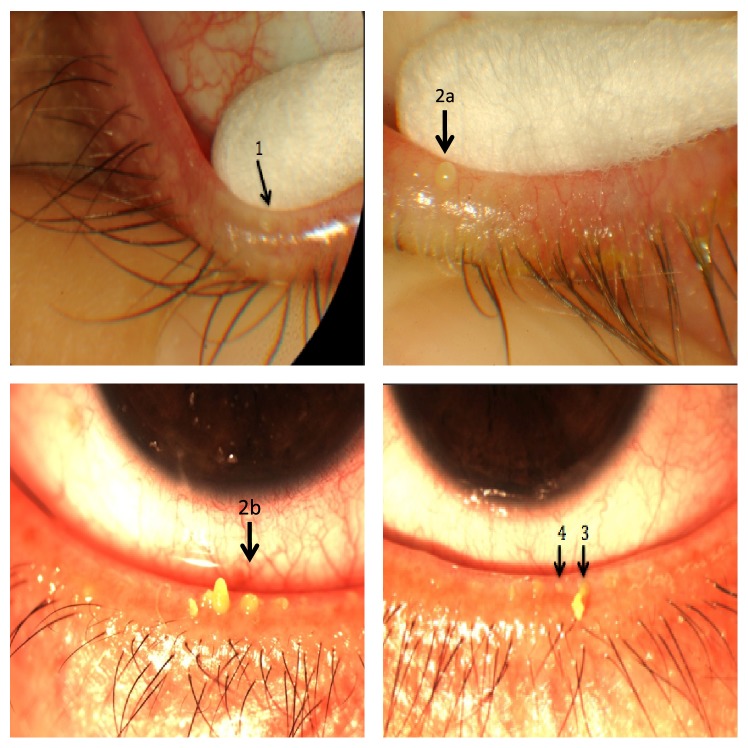
Demonstration of the severity of the COTE as seen by the clinician at the slit lamp, as described in Table 1.

The COTE test, which graded the nature and severity of expressed tarsal gland secretions (Table 1, Figure 1), was devised to evaluate and monitor PB severity. It was performed by the principal investigator at the time of initial evaluation and again after one month. All patients were informed that their competence in performing the PBMP technique would be assessed.

The COTE test is performed at the slit lamp, using a nonpreserved, artificial tear-wetted or warm water-wetted cotton bud (Cb). COTE was performed without topical anaesthesia so that the patient would then have a sense of adequate-enough lid eversion to avoid pain due to inadvertent corneal touch with the Cb. This was emphasised during PBMP instruction, so that it could be subsequently carried out safely at home.

The central lower lid is gently everted digitally by the clinician, usually by the index finger or thumb. Firm anteroposterior pressure is applied to the posterior lid surface between that digit and the Cb at one point along the lid, effectively compressing those tarsal glands. Evaluating the central lid renders the procedure easy, rapid, and pain-free.

COTE grading was documented as grades 1, 2a, 2b, 3, or 4 (Table 1) representing scores of 1, 2, 2.5, 3, or 4 points, respectively. The mean score was used to inform statistical analysis.

### 2.2. PBMP

Patients were then taught the PBMP technique using a plane mirror for myopes and a concave mirror, where necessary, for hypermetropes. PBMP consists of two components requiring a total of 90 seconds per session.

#### 2.2.1. Heat and Massage

**Figure 2:**
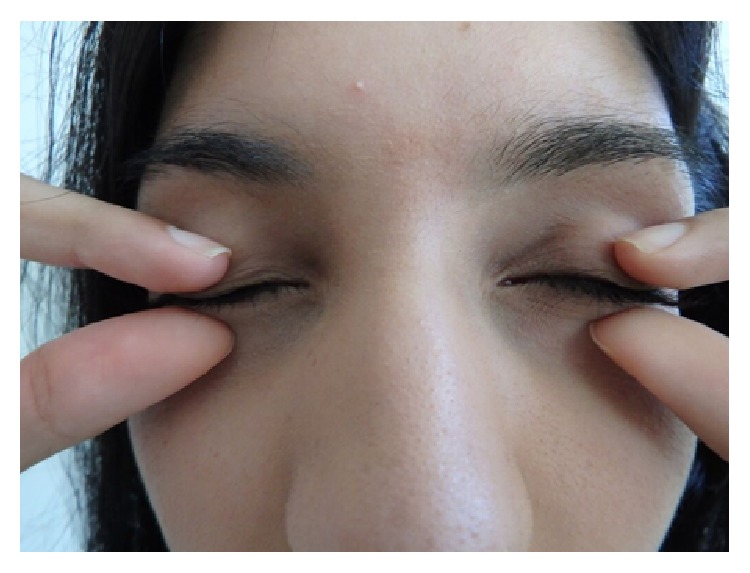
Massage (Part A of PBMP) of the lids. (i) Each patient is asked to close his or her lids under comfortably hot shower water and to massage, gently, both upper lids downwards and both lower lids upwards during those 30 seconds. This is done from a medial to lateral direction. (ii) The patient uses the thumb and index finger of the ipsilateral hand aiming to express the tarsal gland contents towards the lid margins.

Patients were asked to close their eyes for 30 seconds under comfortably hot shower water and to massage simultaneously both upper lids downwards and both lower lids upwards (Figure 2) between ipsilateral thumbs and index fingers. The patient's fingers and thumbs massage along the lids from medial to lateral over the 30-second period.

#### 2.2.2. Lid Scrubs

After showering, patients were instructed to use a warm water-moistened Cb to scrub all four eyelid margins, for 15 seconds per lid (60 seconds).

Each lower and upper lid horizontal margin was divided into five equal theoretical sections. Patients were asked to scrub each of the five sections of the lid margin with the moistened Cb for three seconds, commencing medially and moving laterally.

### 2.3. Lower Lid PBMP

Patients were asked to

**Figure 3:**
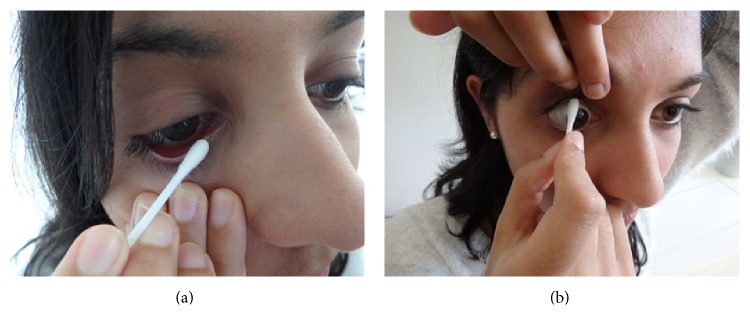
(a) Lid scrubs (Part B of PBMP) of the right lower lid (RLL) using a Cb on the lid margin. (i) The patient's lid and hand have been dried and degreased by wiping with a tissue prior to lid scrubbing. (ii) In this photograph, the patient is applying lid scrubbing to the medial 1/5 of the RLL for 3 seconds. (iii) Note that the lower lid is everted adequately enough to protect the cornea, allowing precise access to its horizontal margin. (v) Note also that the Cb is held at the optimal distance from its tip to optimise patient control of the Cb tip. (b) The “Gorilla Grip” used to evert the right upper lid (RUL) away from ocular surface. (i) Note that the patient is using the Gorilla Grip on the RUL by means of the nondominant hand and middle finger. (ii) The patient is carefully inspecting the RUL with the opposite eye. (iii) The lid margin is consistently kept at least 3 mm from the globe. (v) In this photograph, the patient is carrying out PBMP on the middle 20% of the RUL. Each of the theoretical five segments of each lid takes 3 seconds.

1. hold the Cb not too far from its tip to avoid tip instability, nor too close to reduce tip visibility;
2. use the index or middle finger of the nondominant hand to evert and stabilise the lower lid (Figure 3(a));
3. commence lid scrubs at the most medial section of the lower lid (Figure 3(a));
4. divide the lid into theoretical fifths, allowing 3 seconds of scrubbing for each fifth. The scrubbing was carried out by moving one-fifth of the lid length laterally with each 3-second section of the scrub. By moving the everting finger for every section, it was ensured that the lid was at least at a minimum of 3 mm from the globe, and particularly the cornea, at any time point.

### 2.4. Upper Lid PBMP

Commencing with the medial end of the upper lid, the nondominant index, middle, or ring finger was used to achieve adequate upper lid eversion. Upper lid PBMP was also done in lid-fifths sections.

The “Gorilla Grip” facilitated this step (Figure 3(b)). The patient held the palmar surface of the nondominant hand over the frontal region, just above the eyelid margin, achieving optimal upper lid eversion. The “Gorilla Grip” was further facilitated by gentle traction of the lid margin across the brow, especially for the contralateral upper lid.

The principles of lower lid PBMP (Section 4) were utilised in achieving corneal protection against blunt trauma from the Cb. These were augmented by the patient elevating the chin and having the patient look downwards while the upper lid was being retracted.

### 2.5. PBMP Safety

PBMP safety was achieved by confirming that

1. the patient could see his or her eyelid structures at all times;
2. the lid margin was everted at least 3 mm during PBMP;
3. the patient was aware that the process should be pain-free, as corneal touch is obviated.

### 2.6. PBMP Tuition and Followup

Patients received at least 15 minutes of tuition from the treating Ophthalmologist or supervising orthoptist. Patients were instructed that their competence at PBMP would then be definitively examined at one month.

### 2.7. PB Symptomatology

Resolution of PB symptomatology was recorded on a subjective scale, expressed as a percentage of PB symptom resolution. A range of 0–100% was utilised (ranging from no improvement to complete resolution of symptoms).

## 3. Results

Mean patient age was 59.1 ± 17.1 years. Duration of symptoms was 30.0 ± 18.9 months before commencing PBMP. Tear production assessed using Schirmer's testing with topical anaesthesia revealed 33.3% of patients with ≥9 mm, 44.5% with 4–8 mm, and 22.2% with <4 mm of tear production. Thus, 66.7% had dry or very dry eyes. Eight patients (29.6%) had a history of rosacea.

**Figure 4:**
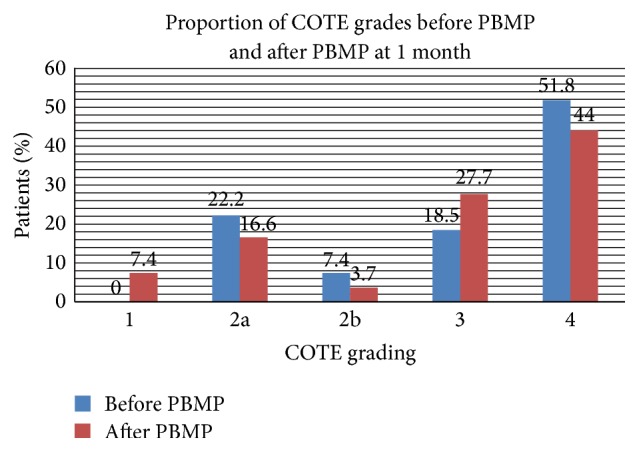
The severity of PB was classified according to COTE criteria.

COTE grading at baseline compared with post-PBMP treatment is summarised in Figure 4. There was a trend towards PBMP improvement, as more eyes with the higher COTE grades improved after treatment. However, there were no significant differences between pre- and posttreatment mean COTE score for all grades at one month (ANOVA *p* = 0.124).

Of note, clear normal tarsal gland secretion (COTE grade 1) was demonstrated in 7.4% of tarsal glands following one month of treatment. Although not statistically significant, the novel appearance of COTE grade 1 was consistent with the trend towards objective improvement.

Reflecting the possible benefit from PBMP, patients reported an average of 77.8 ± 13.5% improvement in their symptoms at one month.

## 4. Discussion

In a major survey conducted by Lemp and Nichols in 2009, 120 Ophthalmologists reported that 37% of their patients suffered from blepharitis [4]. In Lemp's study, 80% of treating Ophthalmologists agreed that improving MGD should be the goal in treating PB, compared with reducing inflammation (19%).

### 4.1. Meibomian Gland Integrity

Some studies [8, 9] have shown that measuring meibomian gland dropout is useful for assessing the baseline integrity of meibomian glands. This has been done using meibography, employing confocal microscopic techniques. While meibography may be a useful research tool, it may not be financially justifiable or clinically useful. By contrast, the COTE test has been useful in assessing, monitoring, and managing MGD.

The International MGD Workshop (2011) recommended documentation of dry eye disease, blink rate, lower lid tear meniscus height, tear osmolarity, tear film breakup time, and Schirmer's test [9]. MGD was further classified by quantifying meibum expressibility and quality [7]. All of these evaluations will help diagnose the disease and its severity, but the value of each one is unclear in terms of diagnosis and management, especially in a busy clinic.

The ideal diagnostic and therapeutic approach should be straightforward enough to be rapidly performed and effective enough to achieve a convincing diagnosis for patients and clinicians.

### 4.2. COTE and MGD-Related PB

In this study, the COTE test was used to assess MGD-related PB clinically. Similar to previous grading systems [7], it utilises meibomian gland expressibility and secretion quality as a surrogate measure of secretory activity.

Although various classification systems exist, the review article of Tomlinson et al. has attempted to correlate symptoms with the number of expressible tarsal glands [7]. This review demonstrated that there was no significant correlation between gland expressibility grading and improvement in symptomatology.

On the other hand, despite our study similarly demonstrating a lack of statistical significance in correlation (*p* = 0.124), there was a clear trend towards improved COTE grading. This was evidenced by a rise in grade 1 COTE findings from 0% pre-PBMP to 7.4% post-PBMP and a reduction in incidence of grade 4 COTE from 51.8% to 44%. Further, our cohort expressed a 77.8% improvement in symptoms, a substantial improvement clearly recognised by the patients.

Nevertheless, the application of COTE has the following limitations.

- Sampling from the central lower lid may not be representative of MGD of the whole lower lid. However, our group never evaluates the upper lid for PB using the COTE test because this would require topical anaesthesia and the conjunctival trauma would likely result in secondary corneal abrasions and patient discomfort.
- Whilst sampling is done at a single time point, secretory function fluctuates from time to time [10].
- Repeated sampling over more gland locations would be helpful, but not practical, due to patient discomfort.
- Tarsal gland activity decreases from the nasal to temporal side of the lid.
- Digital pressure application applied by the Cb may vary because of a change of angle and force of Cb application even by the same examiner and variability of patient eyelid anatomy [10, 11].
- The COTE test may demonstrate multiple grades of severity of PB in the one lid over the application distance of the Cb, despite the fact that the lid is always assessed at its point of maximum accessibility and eversion.

Korb and Henriquez [12] examined lower lids using subjective “forceful” and “gentle” pressures. In their study, utilising patients with normal lids, gentle expression generally produced egress of clear oil only. By contrast, only patients with PB were examined in the PBMP cohort, and grade 1 oil rate was 0% at diagnosis. Thus the data from Korb and Henriquez should not be regarded as being applicable to the pathological scenario of PB.

In a recent study, the number of expressible tarsal glands was correlated with dry eye symptoms using a custom-designed compression device, where the magnitude and duration of force were standardised [10]. However, the likelihood of the force exerted by the compression device across any lid that is physiologically curved both vertically, anteroposteriorly, and mediolaterally is unlikely to be equal at each point. For example, chalazion surgeons, while attempting to achieve lid haemostasis and stability in preparation for chalazion surgery, are familiar with the fact that force exerted under the chalazion clamp varies along the lid. Thus, as the lid tightness increases under the clamp at one point, it is reduced at the others. Thus, standardising the pressure applied is unlikely to be easily achieved over each tarsal gland and may impede monitoring progress of treatment.

As tarsal gland dropout occurs with increasing age due to gland atrophy rather than obstructive MGD, ageing may contribute to poor tarsal gland expressibility [13, 14] as in our cohort with a mean age of 59.1 years.

Further, numerous ophthalmological and systemic factors contribute to MGD. These include contact lens wear, giant papillary conjunctivitis, trachoma, rosacea, psoriasis, discoid lupus erythematosus, Sjögren's syndrome, androgen deficiency, and menopause [15]. Thus, management of non-MGD disease needs to be addressed in managing PB.

### 4.3. The Value of COTE in PB Management

The COTE test has intrinsic advantages.

- The central 20% of the lower lid was the section that was reevaluated, providing an element of reliability.
- The patients were made aware that their own PBMP technique and efficacy would be mandatorily reexamined after 4 weeks of the commencement of treatment. It is possible that this degree of rigour may have resulted in enhanced compliance and subsequently improved symptomatology in patients who had failed PB treatment elsewhere.

The COTE test does have limitations, but it provides a sample of the tarsal gland characteristics and dysfunction. If PB has been diagnosed clinically and patients understand they have MGD as quantified by COTE, they can confidently be offered definitive PBMP.

### 4.4. PBMP Compliance

In 12 years of COTE utilisation, our group has come across numerous patients who have been managed for their PB with a less rigorous technique than PBMP embodies. Simple recommendation of lid heating, followed by lid cleaning with a warm NaHCO_3_ solution or baby shampoo, is inadequate. The current study differs in that mandatory teaching by ophthalmological and orthoptic staff was followed by the “challenge” of an examination of each patient's PBMP technique after a month.

The PBMP study reinforced to each patient the proper technique of PBMP. It was encouraging to note symptom improvement by 77.8%. Considering that some patients had been managed unsuccessfully previously for years, the potential benefit of our PBMP technique was clear.

### 4.5. Symptom/COTE Paradox

Paradoxically, 9 of 54 eyes (16.7%) had worsened COTE grading, but 100% of these patients still reported an improvement in symptoms. It is possible that the residual, still-functioning meibomian glands may have improved their oil output by being rendered functional because of the effective PBMP.

### 4.6. PB Treatment Modalities

Current consensus in the treatment of blepharitis [16] includes the use of “lid hygiene with warm compresses” as the first line. However, even the American Academy of Ophthalmology guidelines [16] do not specify a simple, safe, inexpensive, and effective technique requiring 30 seconds to achieve lid heating as a part of PB management.

Furthermore, this level of “lid hygiene” may not reflect the care intrinsic to our PBMP technique. The timing of each stage of “lid hygiene with warm compresses” may not be rigorously applied. While, in resistant cases, systemic oral antibiotics can be useful [16], none of our patients required antibiotics despite 29% of our cohort demonstrating rosacea.

Alternative medical treatments acting by downregulating inflammatory pathways have been described. Azithromycin may suppress bacterial lipases, while oral omega-3 improves meibum lipid properties [17]. Cyclosporin A immunomodulates conjunctival T-cell activity, while N-acetylcysteine has additional mucolytic and anticollagenolytic properties [17]. Despite their obvious theoretical advantages, attempted tarsal gland evacuation by adequate PBMP is still integral.

Newer treatments with Thermal Pulsation (TP) [18] or Intense Pulse Light Therapy (IPLT) are still under clinical evaluation. The duration of effective TP treatment is unclear, but it has been suggested to be 7–9 months [19, 20]. However, the cost per unit patient treatment may be prohibitive for some patients.

Further, IPLT causes selective photothermolysis of haemoglobin within the telangiectatic vessels of the lid [21]. This leads to lid blood vessel thrombosis, preventing the release of inflammatory mediators [18]. While IPLT can improve the ocular surface [22], a lid rendered ischaemic in this way may well shut down meibomian and goblet cell secretion. By contrast, PBMP attempts to improve the glands that are already functioning and reactivate glands that are grade 4 on COTE.

As the PB waxes and wanes seasonally, along with the patient's stress levels and rosacea and hormonal variations, the patient can increase the PBMP from three times a week to daily as necessary. Again, it requires only 90 seconds of the patient's time per treatment, which is crucial considering that compliance may be an important limiting factor for effective PBMP. The most recent patient protocol for PBMP is attached (Appendix 1) in supplementary material available online at http://dx.doi.org/10.1155/2015/617019.

## 5. Conclusions

The COTE test clearly provides a rapid, objective assessment of the expressibility of the central lower lid tarsal glands in PB. Paradoxically, the COTE test does not correlate highly with PB symptoms.

PBMP appears to be a powerful first-line treatment for PB, as patients experienced a mean symptomatic improvement of 77.8% in their PB. PBMP has been demonstrated in this study to provide a rapid, inexpensive, simple, effective, and safe method of treating PB.

## Supplementary Material

The appendix is a set of instructions given to the patient as part of their PBMP tuition.

## Figures and Tables

**Table 1:** COTE grading system.

Grade	Nature of secretion on compression
1	Clear oil
2a	Easy egress of pus
2b	Slow and difficult egress of pus
3	Thick toothpaste-like secretion (worm-like)
4	Complete blockage of tarsal gland; no egress of secretion visualised
