# Supplementary material for: Utilisation of a Novel Test to Measure Severity and Treatment Efficacy of Posterior Blepharitis
Source: J Ophthalmol. 2015 Aug 12;2015:617019. doi: 10.1155/2015/617019 (PMC4549570; doi:10.1155/2015/617019)
Supplement: Supplementary file 1 — The appendix is a set of instructions given to the patient as part of their PBMP tuition. [file 617019.f1.docx]

**Appendix 1 – Teaching instructions**

**PATIENT MANAGEMENT OF BLEPHARITIS**

Blepharitis, best known as Posterior Blepharitis, is a common and annoying disorder. However, most patients can be helped considerably with careful Blepharitis management.

Ultimately, after the first month of initial treatment, Blepharitis management takes only 90 seconds a day on three mornings a week.

**STEPS (1) and (2)** - **These steps take a total of 30 seconds and are concurrent.**

1. The first two steps are lid heating and massage. They are carried out by turning the temperature up in the morning shower to a little hotter than normal (without burning yourself), and allowing the hot water to run over the closed eyelids.

2. While this lid heating is occurring, you should simultaneously massage your upper lids down and then your lower lids up for 30 seconds. This requires a gentle pincer grip utilising both hands with the index fingers and thumbs of each hand. Start towards the nose side of your eyelids and move every few seconds towards the ear side of your eyelids.

**STEP (3)**

3. After your shower, and using the appropriate mirror, you should clean your lower lids and then your upper lids on one side, followed by the second side, based on the following principles.

Start from the nose side of your lower lid. Hold a single Cotton bud/Q-tip with the index finger and thumb of your right hand, with the tip moistened with fresh warm water straight from the tap. Clean the horizontal margin of the lid from the nose side of the lid to the ear side of the lid in five sections. The lower lid should thus take a total of no more than 15 seconds, spending 3 seconds per section.

**For the lower lid**: At no stage should you massage the inside of the lid with the Cotton bud/Q-tip, nor should you massage the lid below the line of the lashes. At no stage should there be any pain. This only occurs if you touch the cornea. This will not occur if you pull the lower lid slightly out and massage only the lid margin.

**For the upper lid**: Carry out the same process, starting from the nose side and moving to the ear side. Each upper lid should take 15 seconds again. For the upper lid, the GORILLA GRIP should be used. Most people use their index, middle or ring finger to pull the upper lid at least 3 millimetres away from the globe in five sections, lasting 3 seconds each.

**Outcome**: If you do this assiduously, the prognosis is excellent. Within one month, almost everybody improves, with reported symptoms 80% better than they were.

In the first month, Blepharitis management should be carried out on a daily basis.

It is certain that at review after a month, we will be asking you to carry out the process only three times a week for the rest of your cognitively intact life. This will depend on how bad or annoying your symptoms are.

Please remember that the visit at one month is vital, because at that time we will examine your lid massage technique. We will correct any errors that you might have in the technical steps of Blepharitis management.
